# Variations of the origin of superior thyroid artery and its relationship with the external branch of superior laryngeal nerve

**DOI:** 10.1371/journal.pone.0197075

**Published:** 2018-05-10

**Authors:** Meselech Ambaw Dessie

**Affiliations:** Department of Anatomy, School of Medicine, College of Medicine and Health Sciences, University of Gondar, Gondar, Ethiopia; "INSERM", FRANCE

## Abstract

**Introduction:**

The superior thyroid artery, the main atrial supply of neck region, is branched from the external carotid artery as a first branch, but it may also arise from the common carotid artery and its bifurcation. The external branch of superior laryngeal nerve runs parallel to it and later crossing the artery either above or below the upper pole of the thyroid gland.

**Objective:**

This study aimed at evaluating the variations of the origin of superior thyroid artery and its relationship with the external branch of the superior laryngeal nerve.

**Methods:**

A descriptive study was conducted on 43 embalmed cadavers. The anterior triangle of the neck region was dissected bilaterally. The presence or absence of STA and its origin, branching pattern, relationship with the external branch of the superior laryngeal nerve, level of origin in relation to the lamina of the thyroid cartilage and level of carotid bifurcation were observed and recorded.

**Result:**

The superior thyroid artery arises from the external carotid artery in 44.2%, common carotid bifurcation in 27.9% and common carotid artery in 26.7% of cadavers. In one of the cadaver, the superior thyroid artery arises from lingual artery. The origin of superior thyroid artery was significantly associated with its branching pattern and level of common carotid artery bifurcation. The mean distance from the upper pole of the thyroid gland to the level where an external branch of superior laryngeal nerve turns medially from superior thyroid artery was found to be 1.04cm.

**Conclusion:**

The wide range of variations of the superior thyroid artery on its origin and relationship with adjacent structures is a common phenomenon. The clinicians should be aware of those variations.

## Introduction

The superior thyroid artery (STA) is the main arterial supply of the thyroid gland, the upper part of the larynx, muscles and overlying skin of the neck region[1–3]. It is branched from the anterior surface of the external carotid artery (ECA) as a first branch, just below the level of greater cornu of the hyoid bone [1, 3–6]. It descends along the lateral border of thyrohyoid muscle anterolateral to the external branch of superior laryngeal nerve (EBSLN) to reach superior pole of the thyroid gland[3, 7]. Past studies have reported the incidence of origin of the STA from the common carotid artery (CCA) as well as from the bifurcation of the CCA[2, 3]. The artery has the following named branches: infrahyoid, superior laryngeal, sternocleidomastoid and cricothyroid [1, 3, 8].

The STA is involved and has many roles in head and neck surgical procedures [9]. It is frequently used as a recipient vessel in microvascular free tissue grafting, for selective embolization of thyroid and other head and neck tumors, emergency cricothyroidotomy, radical neck dissection, diagnostic and therapeutic catheterization, plastic surgery, reconstruction of the aneurysm, carotid endarterectomy and is a surgical landmark for identifying the EBSLN in thyroid surgery[2, 7, 9, 10]. Surgical treatments of thyroid diseases are the most common clinical practice; consequently, detailed understanding of the blood supply of this gland to the surgeons is immense importance to prevent any alarming hemorrhage[3].

The variations in the origin and distribution pattern of STA are great importance for head and neck surgeons because of its vital relationship to the EBSLN[8, 11]. The EBSLN, which supplies the cricothyroid muscle, runs parallel to STA and later crossing the artery from lateral to medial either above or below the upper pole of the thyroid gland[4, 7]. This muscle tensions the vocal folds to produce high-frequency sound during phonation, protect the airways against aspiration during swallowing, and optimize breathing[11, 12]. The EBSLN comes into play at frequencies above 150 Hz, thus it is particularly involved in producing the high tones. This can be predominantly significant for individuals using their voice professionally[13]. In case of thyroidectomy STA must be ligated and is highly essential to ensure proper hemostasis[8]. Because of its intimate relationship with STA, EBSLN is at risk when the artery is being ligated [8, 11, 14]. The injury of the EBSLN cause palsy of the cricothyroid muscle and alters lower airway protection mechanisms. Thus, EBSLN injury may alter the ability to produce acute sounds and lead to dysphagia, especially with liquids[11]. Clinical symptoms may present as weakness, tightness, and increased effort to speak, increased throat clearing, and vocal fatigue [15, 16]. The severity may vary depending on the voice demands of the person and it is felt that singer and professional voice users, such as lawyers, teachers and broadcasters, are more significantly affected by the subtle changes related to its injury[16, 17]. Even for non-professionals, the perception of an abnormal voice impairs the quality of life and decreases the general health in many ways and affected patients may be unable to shout for help[15]. The incidence of EBSLN injury in patients undergoing thyroidectomy is reported to be up to 58%[13, 15, 18]. One of the earlies reported case goes back to 1935 when the famous opera singer Amelita Galli- Curci suffered from damage to the EBSLN after thyroid surgery. This nerve has since become known as the “nerve of Galli-Curci[16, 17].

The origin of STA and its relation with the EBSLN varies from population to population [4]. Many studies were conducted on the anatomical variations of the origin of the STA and its relationship with EBSLN in different countries of the world, but there were few published reports that show the variations of the STA and its relationship with the EBSLN in Africa particularly in Ethiopia. So this study provides the anatomical variations of the origin of STA and its relationship with EBSLN among Ethiopian people’s based on the cadavers.

## Materials and methods

A descriptive cross-sectional institutional based observational study was conducted on 43 embalmed human cadavers. The study samples were cadavers used for educational purpose in medical colleges of the following institutions: University of Gondar, Bahirdar University, Arbaminch University, Wolaita Sodo University, Wachemo University, Wolkite University, Jimma University, Hawassa University, Dilla University, Arsi University, Hayat Medical College, Bethel Medical College, Sante Medical College and Gamby Medical College. The data were collected from May to November 2017. All the samples have Ethiopian origin. The samples were used by undergraduate medical students for the gross anatomy practical session. If the following structures of the neck region are intact and were not destructed by the students, the cadaver was incorporated in the study: CCA, internal carotid artery(ICA), ECA, STA, facial artery, lingual artery, the vagus nerve, superior laryngeal artery, EBSLN and lobes of the thyroid gland. Cadavers that were macerated by the students and those that were difficult to dissect were excluded from the study. The dissections were accompanied with the cadavers in the supine position and the neck extended. The anterior triangle of the neck region was dissected bilaterally to expose the origin of the STA. The sex of the cadaver, the presence or absence of STA and its origin, branching pattern, level of origin in relation to the lamina of thyroid cartilage, the relation of EBSLN to the STA i.e. distance from the upper pole of the thyroid gland to the level where EBSLN turns medially from STA were observed on both sides and recorded on the checklist prepared for this purpose by the researcher. The photo was taken from the sample for a specific region. A sliding caliper was used to make two measurements on each side of the dissected area, as follows:

1. Distance from upper pole of the thyroid gland to the level where EBSLN turn medially from STA by cm
2. The distance between the origin of the STA and the CCA bifurcation by cm

During the measurement of the distance between those structures, the part of the structures were fixed in position by pins.

In this study, the level of origin of the STA was compared to the lamina of the thyroid cartilage. It was categorized as above the lamina if the STA originates from a carotidal tree at the level above the superior border of the lamina, at the lamina if it is between the upper and lower border of the lamina and below the lamina if it is inferior to the lower border of the lamina. The relation of EBSLN to superior thyroid pole was categorized based on Cernea classification as follows: Type 1 in which the nerve crosses the artery more than 1 cm from the superior pole, Type 2a in which the EBSLN crosses the artery <1 cm and above the superior pole, and in Type 2b in which the EBSLN crosses the artery below the superior pole. The data were entered into epi info 7 version 7.2.1.0 and transferred to SPSS 20 and frequency was calculated for each variable category. The origin of STA was analyzed using a Chi-square test with regard to sex and sides of the cadavers, branching pattern of STA and carotid bifurcation level in relation to the lamina of the thyroid cartilage.

The study was conducted after ethical clearance was obtained from the Institutional Ethical Review Board of the University of Gondar. The study was conducted on the cadavers used by undergraduate and postgraduate students after permission on the federal level.

## Results

**Fig 1:**
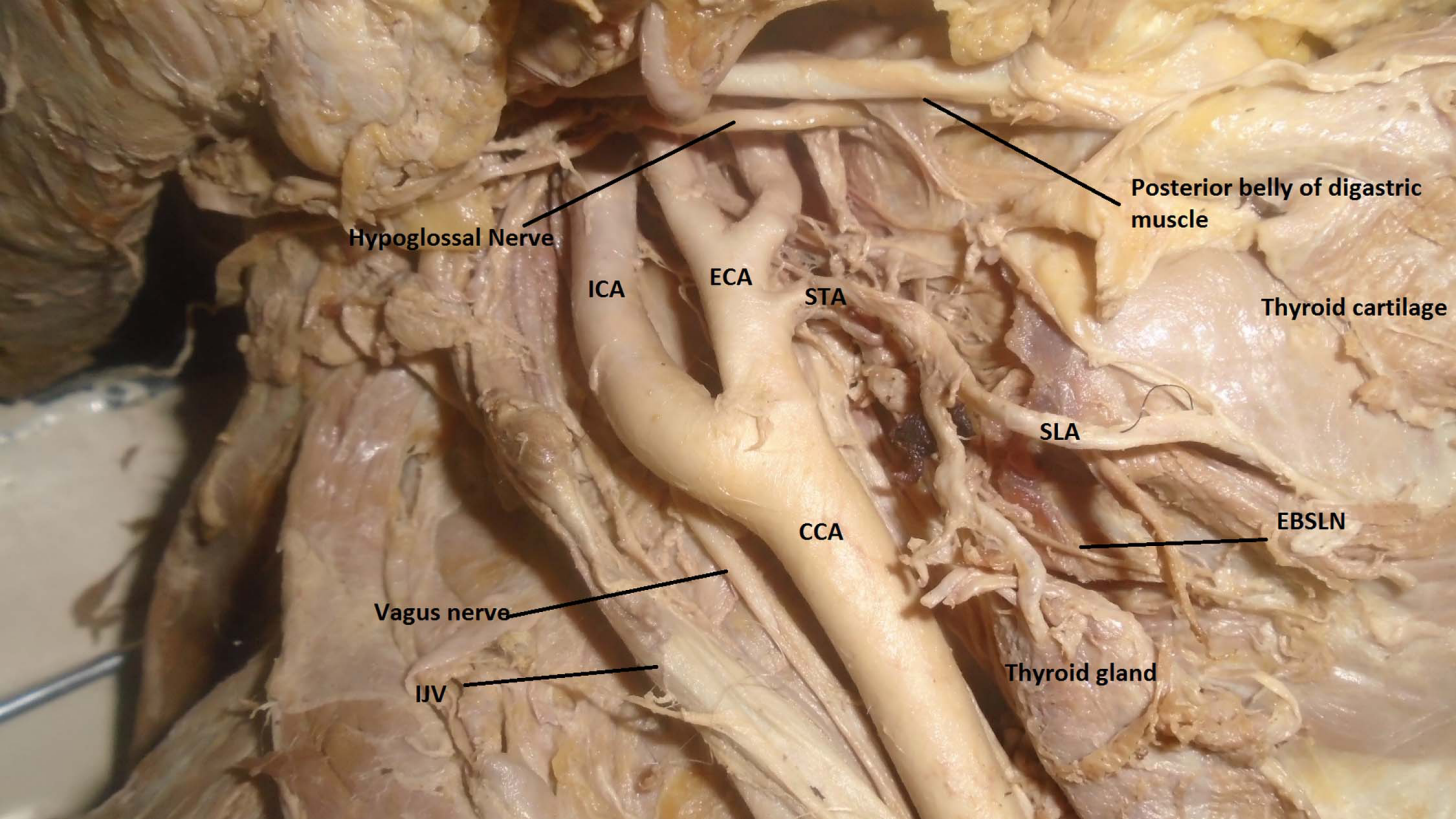
The superior thyroid artery arising from the external carotid artery and EBSLN near the upper pole of the thyroid gland; CCA = Common carotid artery, ECA = External carotid artery, ICA = Internal carotid artery, IJV = Internal jugular vein, SLA = Superior laryngeal artery, EBSLN = External branch of the superior laryngeal nerve.

**Fig 2:**
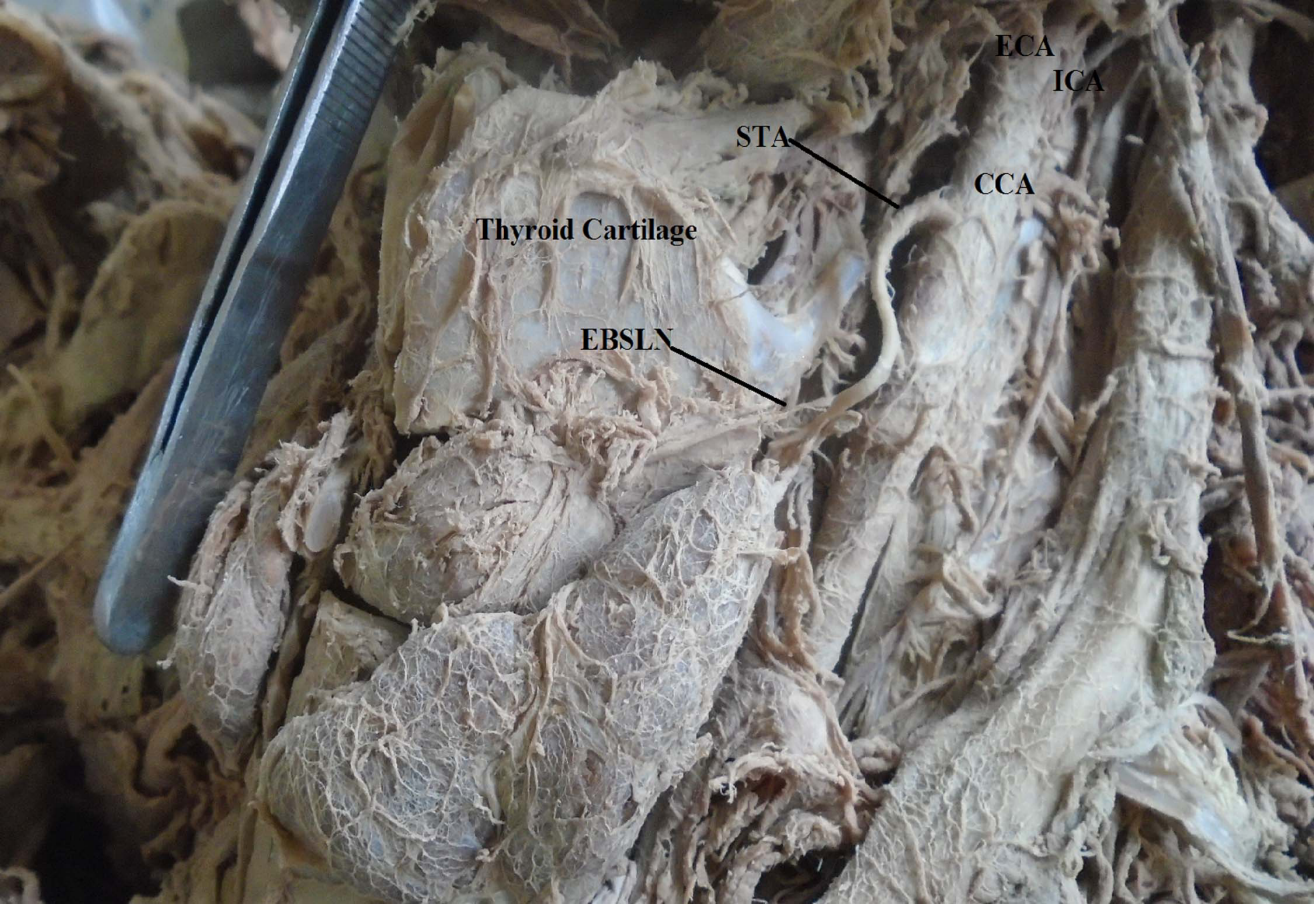
The superior thyroid artery arising from the common carotid artery at the level of the lamina of thyroid cartilage; CCA = Common carotid artery, ECA = External carotid artery, ICA = Internal carotid artery, STA = Superior thyroid artery, EBSLN = External branch of the superior laryngeal nerve.

**Fig 3:**
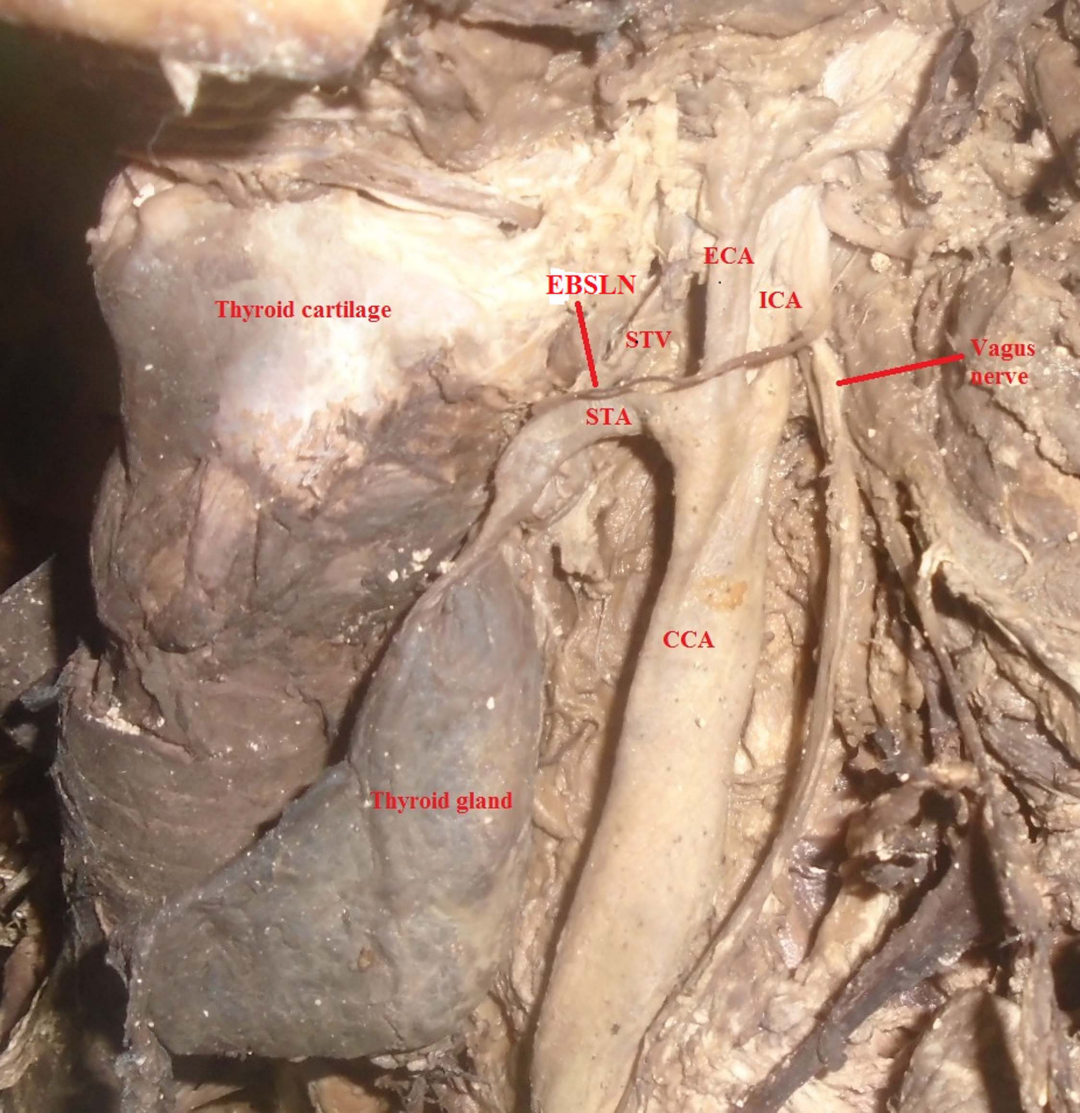
The superior thyroid artery arising from common carotid artery bifurcation at the level of the lamina of thyroid cartilage; CCA = Common carotid artery, ECA = External carotid artery, ICA = Internal carotid artery, STA = Superior thyroid artery, STV = Superior thyroid vein, EBSLN = External branch of the superior laryngeal nerve.

A total of 43 cadavers were included in the study. About 86% (37/43) of the study population were male and 14% (6/43) were female cadavers. The age of the study population was unknown. The STA arises from the ECA in 51.2% and 37.2% of the cases in the right and left sides respectively (Table 1 and Figs 1–3).

**Table 1:** The origin of superior thyroid artery among Ethiopian cadavers.

Site of origin of superior thyroid artery	Right side (n = 43) (%)	Left side (n = 43) (%)	Total (n = 86) (%)
External carotid artery	22(51.2%)	16 (37.2%)	38(44.2%)
Common carotid artery	8(18.6%)	15 (34.9%)	23(26.7)
Common carotid artery bifurcation	12 (27.9%)	12 (27.9%)	24(27.9)
Other (Lingual artery)	1(2.3%)	-	1(1.2%)

The STA was branched independently from the carotid tree in 94.2% (81/86), commonly branched with the thyrolingual and linguofacial trunk in 2/86(2.3%) for each (Table 2).

**Table 2:** Branching pattern of superior thyroid artery from the carotid tree.

Branching pattern	Side of the body		Total
	Right	Left	
Independently branched	90.7%,	97.7%	94.2%
Branched from linguofacial trunk	2.3%	2.3%.	2.3%
Branched from thyrolingualtrunk	4.7%	-	2.3%

The origin of STA was significantly associated with its branching pattern with a p-value of 0.007. About 75% (3/4) of the arteries which were branched from a common trunk arises from ECA, 2/3 are on the right side. The remaining 25% (1/4) of the artery from the common trunk arises from the right side of the carotid bifurcation.

**Fig 4:**
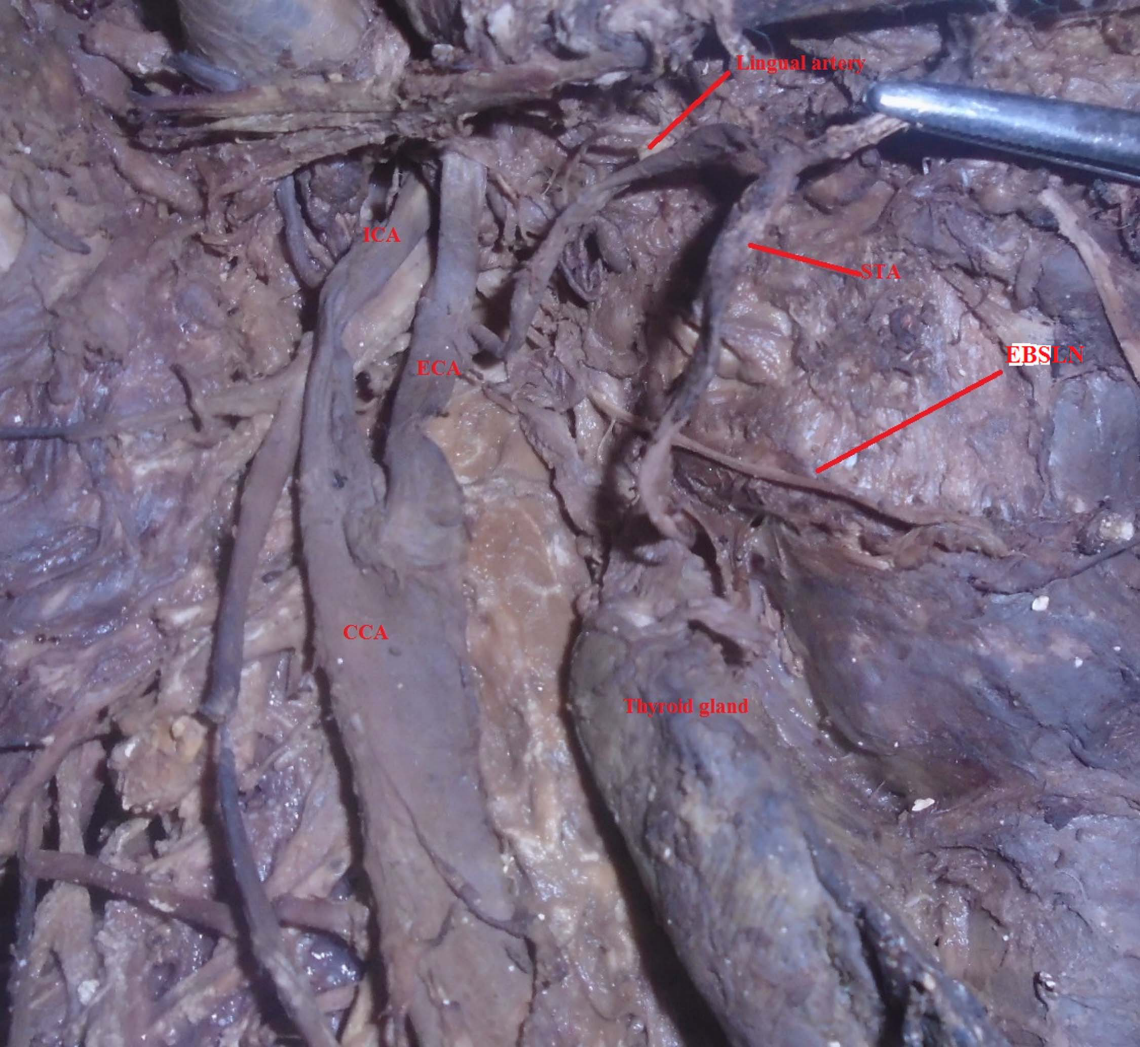
STA arising from the lingual artery and EBSLN passing posterior to it after removal of posterior belly of digastric and hypoglossal nerve; CCA = Common carotid artery, ECA = External carotid artery, ICA = Internal carotid artery, STA = Superior thyroid artery, EBSLN = External branch of superior laryngeal nerve.

In the right side of one cadaver, STA arise from the lingual artery (Fig 4). This lingual artery arises from the ECA at the level of the upper border of the thyroid cartilage, 2cm above the CCA bifurcation. Then it ascends superiorly towards the inferior border of the mandible posterior to the hypoglossal nerve and the posterior belly of the digastric muscle. Inferior to the submandibular gland at the level of the body of hyoid bone, the STA arises from the lingual artery and descends to the upper pole of thyroid gland anterior to the hypoglossal nerve.

The level of origin CCA bifurcation was significantly associated with the origin of STA (p-value = 0.00). In the arteries which arise from the CCA, 90.3% of the CCA bifurcates above the lamina of the thyroid cartilage. In 86.8% of the arteries which arise from ECA, the CCA bifurcates at the lamina. The right and left STAs were symmetrical based on their origin from the carotidal tree in 22/43(51.2%) of the study subject. The mean distance between the origin of STA and CCA bifurcation for the arteries which arise from ECA was 0.6cm with a 0.5cm, 0.3cm and 0.5cm median, mode and standard deviation respectively. The minimum distance from the origin of STA to the CCA bifurcation was 0.1cm and the maximum was 3 cm. The distance from the origin of STA and CCA bifurcation for the arteries which arise from CCA was found to range from 0.2cm to 3.5cm with 1.1cm, 0.7cm, 0.3cm and 1cm mean, median, mode and standard deviation respectively. The minimum distance from the origin of STA to the CCA bifurcation was 0.2cm and the maximum was 3.5cm. There was no statically significant relation between the origin of STA and the sex and side of the cadavers.

In relation to laminae of the thyroid cartilage, the level of origin of STA was above the laminae of thyroid cartilage in 24(55.8%) of the right side, 20(46.5%) of the left side and 51.2% (44/86) of both sides. The STA arise at the level of the lamina in 18(41.9%) of the right side, in 23(53.5%) of the left side, in 41(44.7%) of both sides.

The STA was observed closely related to EBSLN on both sides (Table 3). The mean distance from the upper pole of the thyroid gland to the level where EBSLN turn medially from STA was found to be 1.04cm with the mode of 1.00cm, median 1.0cm, standard deviation 0.5cm, minimum 0.3 and maximum of 3.0cm.

**Table 3:** Relation of external branch of the superior laryngeal nerve to the superior thyroid artery according to Cernea’s classification.

	Right side	Left side	Total
Type 1	24(55.8%)	25(58.1%)	49(57.0%)
Type 2a	18(41.9%)	17(39.5)	35(40.7%)
Type 2b	1(2.3%)	1(2.3%)	2(2.3%)

## Discussions

In the standard anatomical, surgical and radiological textbooks, the STA is considered to have a relatively constant origin from the anterior surface of ECA[19, 20]; but many studies have reported wide variations in the origins of the STA[2, 8, 21–23]. The STA frequently arises from the ECA just above the carotid bifurcation. It may also arise from the CCA, from CCA bifurcation or as a common trunk with the lingual and facial branches of ECA[24]. Studies have also reported the origin of STA from the ICA and subclavian artery (SCA) [2, 6, 24]. The CCA and ICA develop from the third aortic arch and the ECA sprouts cranially as a new vessel from the third aortic arch and the variations might be related to changes in the transformation of the aortic arch system in the embryo [7, 10, 21].

In most of the previous studies, the STA arises primarily from ECA, which ranges from 60–80.4% [2, 21, 22, 24]. The common carotid bifurcation was a primary origin of STA in a study done in Spain by Vazquez et al, which accounts 49%. In this study, STA arises from ECA in 23% of cases[22]. The minimum incidence of STA originating from CCA bifurcation was 2.2% as reported by Kevin W. Onget in Kenya[24]. A study in India by Joshi et al shows 1.51% of STA arise from CCA[2](Table 4).

**Table 4:** Comparison of the origin of superior thyroid artery from different pieces of literature and the current study.

Origin of STA	Right	Left	Total	First Author	Study area	Population
External carotid artery	20 (60.61)	24 (72.73)	44(66.67)	Abhijeet Joshi	India	Asian
	9 (60%)	9 (60%)	18 (60%)	Manjunath C S		
	10/14(71.5%)	8/11(72.5%)		Pankaj Gupta		
	-	-	6(20.0%)	Sung-Yoon Won	Korea	
	-	-	48(23%)	Teresa Vazquez	Spain	European
			-	Ozgur Z	Turkey	
	413(64.5%)	254(39.7%)	-	Kaan Esen		
			39%	Natsis	Greek	
	-	-	80.4%	Kevin W. Ongeti	Kenya	African
	22(51.2%)	16 (37.2%)	38(44.2%)	Current study	Ethiopia	
Common carotid artery	01 (3.03)	-	01 (01.51	Abhijeet Joshi	India	Asian
	1 (6.66%)	4 (27.7%)	5 (16.66%)	Manjunath C S		
	¼(7.14%)	-	-	Pankaj Gupta		
			12 (40.0%)	Sung-Yoon Won	Korea	
	-	-	55(27%)	Teresa Vazquez	Spain	European
			35%	Ozgur Z	Turkey	
	90(14.1%)	226(35.3%)	-	Kaan Esen		
			12%	Natsis	Greek	
	-	-	10.9	Kevin W. Ongeti	Kenya	African
	8(18.6%)	15 (34.9%)	23(26.7)	Current study	Ethiopia	
Common carotid bifurcation	12 (36.36)	09 (27.27)	21 (31.81)	Abhijeet Joshi	India	Asian
	5 (33.34%)	2 (13.34%)	7 (23.34	Manjunath C S		
	3/14 (21%	2 (18.5%)		Pankaj Gupta		
			12 (40.0)	Sung-Yoon Won	Korea	
	-	-	102(49%)	Teresa Vazquez	Spain	European
			40%	Ozgur Z	Turkey	
	131(20.5%)	148(23.1%)	-	Kaan Esen		
			49%	Natsis	Greek	
	-	-	2.2%	Kevin W. Ongeti	Kenya	African
	12 (27.9%)	12 (27.9%)	24(27.9)	Current study	Ethiopia	

In this study, the STA arises independently from the carotidal tree in 94.2% and as a common trunk i.e. thyrolingofacial in 2.3% and thyrolingual in other 2.3%. The STA originated independently in 90% of cases based on the report by Ozgur et al[9]. The STA arise from the thyrolingual trunk in 0.6% as reported by Vazque et al in Spain and in 2.5% as reported by Ozgur et al from Turkey[9, 22]. In studies conducted in Kenya by Ongeti et al, in Spain by Vazque et al and in Turkey by Ozgur et al, the STA originated from the linguofacial trunk in 6.5%, 0.3% and 7.5% respectively[9, 22, 24]. The origin of the STA from CCA was associated with high carotid bifurcation and origin from ECA was associated with low carotid bifurcation. This finding was consistent with other studies previously[10, 24]. There was the asymmetric origin in 48.8% of cases. Asymmetric origin was reported in 6.5% of cases by Ongeti et al[24].

In relation to laminae of the thyroid cartilage, the level of origin of STA was above the laminae of thyroid cartilage in 51.2% and at the lamina in 44.7% of cases. The level of origin of STA was above the thyroid cartilage in 86.36% and at the same level of thyroid cartilage in 13.64% of cases based on the study by Joshi et al[2].

In the current study, the distance between the origin of the STA from the CCA and the CCA bifurcation was 0.2cm to 3.5cm and the distance between the origins of STA from ECA to the carotid bifurcation was 0.1cm to 3cm. According to Vazquez et al for the STA arise from CCA the distance from the origin to the carotid bifurcation was between 0.1 and 2.1 cm and for the arteries which arise from ECA it was 0.1 to 1.5 cm[22].

The EBSLN crossed the STA more than 1 cm or at a 1cm distance from the upper pole of the thyroid gland(Type 1) in 75%, 72.73%, and 60% of cases according to Magoma et al, Joshi et al, and Estrela et al respectively[2, 4, 11]. In the present study, the EBSLN cross the STA at 1 cm or more than 1cm from the upper pole of the thyroid gland in 57.0% of cases.

The EBSLN crossed the STA less than 1cm above the upper pole of the thyroid gland(Type 2a) in 25%, 27.27%, 17% and 40.7% based on the studies of Magoma, Joshi, Estrela and the present study respectively[2, 4, 11].

The EBSLN cross STA below the upper pole of the thyroid gland(Type 2b) in 20% according to Estrela[11]. In this study, 2.3% of nerve crosses the STA below the upper pole of the thyroid gland.

Although it is not assessed by this study, the relation of the EBSLN to the inferior constrictor muscle is also vital in the identification of the EBSLN. Based on Freidman classification there are three categories: Type 1 in which the EBSLN runs its whole course superficially or laterally to the inferior constrictor muscle, Type 2 in which the EBSLN penetrates the muscle in its lower portion and Type 3 in which the nerve dives under the superiormost fibers of the muscle[16]. The present study provides a baseline for the variations of the origin of STA and its relationship with the EBSLN in Ethiopia. And further study is needed to elaborate the relationship between the EBSLN and inferior constrictor muscle among Ethiopian community.

## Conclusions

The wide range of variations of the STA on its origin, course, branching patterns and relationship with adjacent structures is a common phenomenon. Thus the knowledge of these variations is critical for physicians and could help to avoid serious complications during thyroid surgeries, tracheostomy, radiological examination, laryngeal surgeries and microvascular surgeries.

## Supporting information

S1 File Epi Info data for variations origin of STA and its relationship with EBSLN. (MDB)

S2 File Epi Info data for variations origin of STA and its relationship with EBSLN. (PRJ)

## Abbreviations

STA: Superior thyroid artery
ECA: External carotid artery
CCA: Common carotid artery
EBSLN: External branch of superior laryngeal nerve
ICA: Internal carotid artery
SCA: Subclavian artery
